# Retinol Binding Protein 4 Concentrations Relate to Enhanced Atherosclerosis in Obese Patients with Rheumatoid Arthritis

**DOI:** 10.1371/journal.pone.0092739

**Published:** 2014-03-20

**Authors:** Patrick H. Dessein, Linda Tsang, Gavin R. Norton, Angela J. Woodiwiss, Ahmed Solomon

**Affiliations:** 1 Cardiovascular Pathophysiology and Genomics Research Unit, School of Physiology, Faculty of Health Sciences, University of the Witwatersrand, Johannesburg, South Africa; 2 Milpark Hospital, Johannesburg, South Africa; 3 Department of Rheumatology, Charlotte Maxeke Johannesburg Academic Hospital, Faculty of Health Sciences, University of the Witwatersrand, Johannesburg, South Africa; University of Barcelona, Faculty of Biology, Spain

## Abstract

**Background:**

Retinol binding protein 4 (RBP) enhances metabolic risk and atherogenesis. Whether RBP4 contributes to cardiovascular risk in rheumatoid arthritis (RA) is unknown.

**Methods:**

We assessed RBP4 concentrations and those of endothelial activation molecules including E-selectin, vascular cell adhesion molecule-1, intercellular adhesion molecule-1 and monocyte chemoattractant protein-1 by ELISA, and the common carotid artery intima-media thickness (cIMT) and carotid artery plaque by ultrasound in 217 (112 black and 105 white) patients with RA. Relationships were identified in potential confounder and mediator adjusted mixed regression models.

**Results:**

RBP4 concentrations were associated with systolic and mean blood pressure, and those of glucose and E-selectin (partial R = −0.207 (p = 0.003), −0.195 (p = 0.006), −0.155 (p = 0.03) and −0.191 (p = 0.007), respectively in all patients); these RBP4-cardiovascular risk relations were mostly reproduced in patients with but not without adverse traditional or non-traditional cardiovascular risk profiles. RBP4 concentrations were not associated with atherosclerosis in all patients, but related independently to cIMT (partial R = 0.297, p = 0.03) and plaque (OR (95%CI) = 2.95 (1.31–6.68), p = 0.008) in those with generalized obesity, as well as with plaque in those with abdominal obesity (OR (95%CI) = 1.95 (1.12–3.42), p = 0.01).

**Conclusion:**

In the present study, RBP4 concentrations were inversely associated with metabolic risk and endothelial activation in RA. This requires further investigation. RBP4 concentrations were related to enhanced atherosclerosis in patients with generalized or/and abdominal obesity.

## Introduction

Retinol binding protein 4 (RBP4) was characterized in 1968 for its transporting role of retinol from storage sites in the liver to extrahepatic tissues [1]. During the beginning of this century, RBP4 was discovered to be an adipokine in that it is produced by adipocytes, induces gluconeogenesis by stimulating phosphoenol-pyruvate carboxykinase in the liver and impairs peripheral and hepatic insulin sensitivity [2]–[5]. Several RBP4 gene variants are associated with adiposity, the predisposition to visceral accumulation of adipose tissue, insulin secretion or/and insulin resistance and type 2 diabetes [5], [6]. Visceral obesity [7] and liver fat content [8] is associated with high circulating RBP4 concentrations, which relate to metabolic risk factors [3], [5].

A recent cellular study showed that RBP4 consistently stimulates the expression of inflammatory molecules in human retinal capillary and umbilical vein endothelial cells [9]. High RBP4 concentrations are associated with increased atherosclerosis [10] and incident coronary event rates [11].

Several adipokines other than RBP4 and including adiponectin [12], leptin [13] and resistin [14] can participate importantly in the pathophysiology of rheumatoid arthritis (RA), a prototypic inflammatory disease. In this regard, RBP4 concentrations are also associated with those of inflammatory markers, and successful lifestyle intervention in obese subjects results in reduced RBP4 concentrations that are closely related not only to decreased insulin resistance, triacylglycerol levels and blood pressure but also reduced systemic inflammation [15]. A recent investigation revealed that treatment with tumor necrosis factor-α blockade reduces RBP4 concentrations in ankylosing spondylitis, another inflammatory disorder [16].

Patients with RA sustain a markedly enhanced risk of cardiovascular disease (CVD) that is effectuated by adverse conventional cardiovascular risk factors, high-grade inflammation and genetic determinants [17]–[20]. Nevertheless, atherogenesis in RA remains inadequately elucidated and current recommendations on CVD risk stratification reportedly have important limitations [18], [21], [22]. It is in this context that the need for identifying novel biomarkers of enhanced cardiovascular risk in RA has been raised [18], [21].

The presence of rheumatic disease can impact on the production as well as the relationships of adipokines with cardiometabolic risk and atherogenesis [23]–[25]. Lupus alters the effects of leptin on lipid metabolism and atherogenesis [23]. Whereas adiponectin is well recognized for its protective effects against cardiovascular risk in the population at large, adiponectin concentrations associate independently and paradoxically with high blood pressure in black Africans with but not without RA [24], [25]. A paradoxically positive adiponectin-endothelial activation relationship was also documented in white patients with RA [25]. The potential role of RBP4 in atherogenesis and CVD risk stratification in RA has, to our knowledge, not been investigated.

In the present study, we measured RBP4 concentrations, surrogate markers of enhanced early atherogenesis comprising E-selectin, vascular cell adhesion molecule-1 (VCAM-1), intercellular adhesion molecule-1 (ICAM-1) and monocyte chemoattractant protein-1 (MCP-1), and common carotid artery intima-media thickness and carotid artery plaque by ultrasound in both black and white patients with RA. We examined the independent relationships of RBP4 concentrations with cardiometabolic risk, endothelial activation and carotid atherosclerosis.

## Materials and Methods

### Patients

The present study was conducted according to the principles outlined in the Helsinki declaration. The Human Research Ethics Committee (Medical) from the University of the Witwatersrand in Johannesburg, South Africa approved the protocol (approval number: M06-07-33). Participants gave informed, written consent. This investigation forms part of an ongoing study on cardiovascular risk in RA as previously reported [26]–[28]. Two hundred and seventeen consecutive African patients (112 black and 105 white) that met the 1988 American College of Rheumatology and 2010 American College of Rheumatology/European League Against Rheumatism criteria for RA [29], [30] were enrolled. All invited participants agreed to participate. Each patient used disease modifying agents for rheumatic disease (DMARD) at the time of the study. The mean (SD) number of DMARD employed was 2.4 (0.9) and these included methotrexate, chloroquine, leflunomide, sulphasalazine, azathioprine, tetracycline, cyclophosphamide, penicillamine, tumor necrosis factor-α inhibitors and rituximab in 84.8, 66.8, 29.5, 20.3, 14.8, 10.6, 3.7, 2.8, 3.7 and 1.2% of patients respectively. Prednisone was used by only 2.3% of participants. Data were missing in fewer than 5% of any of the recorded characteristics.

### Assessments

We recorded demographic features and lifestyle factors. Height, weight and waist and hip circumference were measured using standard approaches. The body mass index (BMI) was calculated and abdominal obesity and fat distribution were estimated by waist circumference and waist-height ratio, and waist-hip ratio respectively. For the purpose of this study, generalized and abdominal obesity were diagnosed in patients with a BMI of >29.9 kg/m2 and National Cholesterol Education Program metabolic syndrome waist circumference criterion [31], respectively. We recorded disease duration and rheumatoid factor status. Disease activity was assessed by the Clinical Disease Activity Index (CDAI) [32]. Extra-articular manifestations included the current or previously recorded (hospital record review) presence of pericarditis, pleuritis, Felty's syndrome, cutaneous vasculitis, neuropathy, scleritis or episcleritis, retinal vasculitis, glomerulonephritis, vasculitis affecting other organs, amyloidosis, keratoconjunctivitis sicca, xerostomia, Sjogren's syndrome, pulmonary fibrosis, bronchiolitis obliterans organizing pneumonia, cervical myelopathy, subcutaneous nodules and rheumatoid nodules in other locations [33]. C-reactive protein concentrations were determined using immunoturbidimetric methods. Standard laboratory blood tests of erythrocyte sedimentation rate, renal and liver function, hematological parameters, lipids and glucose were performed. The glomerular filtration rate was estimated using the Modification of Diet in Renal Disease equation [34]. Cardiovascular drugs included antihypertensive agents and glucose and lipid lowering drugs.

Recorded metabolic risk factors included systolic, diastolic and mean blood pressure, lipid concentrations and ratios, and glucose levels. Hypertension was defined as an average systolic blood pressure ≥140 or/and diastolic blood pressure ≥90 mmHg or/and current use of antihypertensive medications. Dyslipidemia was diagnosed when the atherogenic index, i.e. the cholesterol-HDL cholesterol ratio was >4 and proatherogenic non-HDL cholesterol concentrations were calculated by subtracting HDL cholesterol from total cholesterol concentrations [35], [36]. Diabetes was identified as the use of glucose lowering agents or a fasting plasma glucose ≥7 mmol/l, and raised fasting glucose when the respective concentrations was ≥5.6 mmol/l [31]. As reported previously, we also measured interleukin-6 (IL-6) concentrations [37] and those of 3 other adipokines including adiponectin that comprises different isoforms [25], resistin [26] and leptin [27].

We measured endothelial activation molecule concentrations including those of soluble E-selectin, VCAM-1, ICAM-1 and MCP-1 using a solid-phase sHS, R & D Systems, Inc., Minneapolis, MN, USA). Their lower detection limits were 0.009 ng/l, 0.6 ng/l, 0.096 ng/l and 5.0 pg/ml respectively; their inter- and intra-assay coefficients of variation were 7.9 and 5.8, 7.0 and 3.1, 5.5 and 4.6 and 5.7 and 5.8 respectively.

BAS (see acknowledgement) and AS performed the carotid artery ultrasound measurements in private and public healthcare patients, respectively. Both operators obtained images of at least 1 cm length of the distal common carotid arteries for measurement of the intima-media thickness of the far wall from an optimal angle of incidence defined as the longitudinal angle of approach where both branches of the internal and external carotid artery are visualized simultaneously [38] and with high resolution B-mode ultrasound (Image Point, Hewlett Packard, Andover, MA, USA and SonoCalc IMT, Sonosite Inc, Bothell, Wash, USA used by BAS and AS, respectively) employing linear array 7.5 MHz probes. The details of the methodology used by BAS were reported previously [39]. The equipment used by AS involves the application of a unique semi-automated border detection program that was previously found to provide highly reproducible results [38]. The intima-media thicknesses in the left and right common carotid artery were measured and the cIMT was defined as the mean of these. Carotid artery plaque was defined as a focal structure that encroaches into the arterial lumen of at least 0.5 mm or 50% of the surrounding intima-media thickness value, or demonstrates a thickness of >1.5 mm as measured from the media-adventitia interface to the intima-lumen interface [40]. Both operators were blinded to the cardiovascular risk profiles of the patients. Repeat ultrasound examinations by both operators on 23 patients revealed Spearman correlations between repeat cIMT measurements of 0.983 and 0.956 for BAS and AS, respectively, and the correlation between measurements made by BAS and AS was 0.926. Both operators identified carotid artery bulb or/and internal carotid artery plaque in 11 of these 23 patients with full agreement.

RBP4 concentrations were measured in serum using a solid-phase sandwich ELISA (Quantikine HS, R&D Systems, Inc., Minneapolis, MN, USA). The lower detection limit was 0.048 mM/l and the inter- and intra-assay coefficients of variation were 7.2 and 6.9% respectively.

### Data analysis

Dichotomous variables are expressed as proportions and continuous variables as mean (SD) or median (interquartile range) when non-normally distributed. Non-normally distributed characteristics were also logarithmically transformed prior to their inclusion in multivariable statistical analysis.

Associations of age at disease onset or at the time of the study and sex and population grouping with RBP4 concentrations were assessed by entering the respective characteristics together in single mixed regression models. Associations of other baseline characteristics with RBP4 concentrations were evaluated in models with adjustment for demographic characteristics that included age at the time of the study and not age at disease onset since the latter was less strongly related to RBP4 concentrations.

The independent relations of RBP4 concentrations with metabolic risk factors were assessed in demographic characteristic, alcohol use, glomerular filtration rate (potential confounders or/and determinants identified in previous analysis) and cardiovascular drug use adjusted mixed linear regression models; waist-hip ratio was additionally forced into these models. The independent relations of RBP4 concentrations with endothelial activation and cIMT and plaque were determined in Framingham score (calculated from age, sex and major conventional risk factors), race, alcohol use, waist-hip ratio glomerular filtration rate, and systemic inflammation (C-reactive protein and interleukin-6 concentrations) adjusted mixed (linear or logistic as appropriate) regression models.

Patients with RA that experience conventional major risk factors or severe disease are reportedly at high risk of cardiovascular disease. Inflammation also directly increases cardiovascular risk and contributes to the atherogenic effects of conventional cardiovascular risk factors. Additionally, age is an established independent risk factor for RA as well as cardiovascular disease, and obesity contributes significantly to an increased 10-year cardiovascular disease event probability in RA patients [41]. Obesity also influences RBP4 production in non-RA subjects. For these reasons together with our recent experience with adipokine metabolism in RA [42], additional sensitivity analysis was performed with re-evaluation of the respective associations in various subgroups. When appropriate, patients were categorized in subgroups based on median values; accordingly, as only 27 patients had diabetes. In view of the small number of patients that were male or rheumatoid factor positive or had extra-articular features, sensitivity analysis in subgroups based on the presence or absence of these characteristics was not performed. Also, with regard to acute phase responses, as the erythrocyte sedimentation rate is more closely associated to cardiovascular disease than C-reactive protein concentrations in RA, patients were stratified based on their erythrocyte sedimentation rate.

Statistical computations were made using the GB Stat program (Dynamic Microsystems, Inc, Silverspring, Maryland, USA) and SAS software, version 9.1 (The SAS Institute, Cary, NC). Significance was set at p<0.05.

## Results

### Patient Characteristics

The demographic features, lifestyle factors, anthropometric measures, RA characteristics, systemic inflammatory markers including C-reactive protein and interleukin-6 concentrations and leukocyte counts, glomerular filtration rate and use of antirheumatic and cardiovascular agents in the present cohort were previously reported. The median (interquartile range) erythrocyte sedimentation rate, and alanine transferase and aspartate transferase concentrations were 13 (5–28) mm/hr, and 22 (17–28) and 25 (20–30) u/l, respectively. Conventional metabolic risk factors, adipokine and endothelial activation molecule concentrations, and cIMT and plaque prevalence are shown in Table 1. Generalized and abdominal obesity were present in 58.7 and 34.3, and 43.9 and 16.3 and % of black and white patients, respectively. Only 7 patients had previously diagnosed established cardiovascular disease that included cerebrovascular disease, myocardial infarction and peripheral arterial disease in 5, 1 and 1 of the cases, respectively.

**Table 1:** Conventional metabolic risk factors, adipokines, endothelial activation and carotid atherosclerosis in activation and carotid atherosclerosis in 217 RA patients.

**Conventional metabolic risk factors**	
Hypertension (%)	62.2
Total cholesterol-HDL cholesterol ratio>4	18.4
Diabetes (%)	12.4
Blood pressure values	
Systolic blood pressure (mmHg)	135 (22)
Diastolic blood pressure (mmHg)	83 (13)
Mean blood pressure (mmHg)	118 (18)
Lipid values	
Total cholesterol (mM)	4.8 (1.0)
HDL cholesterol (mM)	1.54 (1.30–1.89)
Total cholesterol-HDL cholesterol ratio	3.2 (1.0)
LDL cholesterol (mM)	2.7 (0.8)
Non HDL cholesterol (mM)	3.1 (2.6–3.8)
Triacylglycerols (mM)	1.0 (0.8–1.4)
Triacylglycerol-HDL cholesterol ratio	0.7 (0.5–1.0)
Glucose (mM)	4.8 (4.5–5.2)
**Adipokines**	
Leptin (pM)	638 (342–1159)
Adiponectin (μg/ml)	7.36 (4.82–12.19)
Leptin-adiponectin ratio	86.7 (70.9–95.1)
Resistin (nM)	2.71 (1.82–4.25)
Retinol binding protein 4 (μM)	1.78 (0.09)
**Endothelial activation**	
E-selectin (ng/ml)	39.0 (18.6)
VCAM (ng/ml)	833 (668–1041)
ICAM-1 (ng/ml)	274 (211–352)
MCP-1 (pg/ml)	424 (265–679)
**Carotid atherosclerosis**	
cIMT (mm)	0.709 (0.109)
Plaque (%)	40.6

Mean (standard deviation (SD)), median and interquartile range or proportions are shown. HDL  =  high density lipoprotein, LDL  =  low density lipoprotein, VCAM  =  vascular cell adhesion molecule, ICAM  =  intercellular adhesion molecule, MCP  =  monocyte chemoattractant protein, cIMT  =  common carotid artery intima-media thickness.

### Associations between Baseline Characteristics and RBP4 Concentrations

As given in Table 2, age at the time of the study as well as age at RA onset and alcohol use were associated with high, and black ethnicity and the glomerular filtration rate with low RBP4 concentrations. In univariate analysis, the mean (SD) RBP4 concentrations were 1.47 (0.88) and 2.13 (0.92) mM/l in black and white patients with RA (p<0.0001).

**Table 2:** Associations of baseline characteristics with retinol binding protein 4 concentrations.

Characteristics	Partial R	p
Age at disease onset	**0.157**	**0.02**
Age at study time	0.169	0.01
Female	0.025	0.7
Race (white = 1; black = 2)	**−0.330**	**<0.0001**
Smoking current	0.109	0.1
Exercise	−0.079	0.3
Alcohol use	**0.160**	**0.02**
Body mass index	0.027	0.7
Waist circumference	−0.013	0.9
Waist-hip ratio*	0.061	0.4
RA duration	−0.050	0.5
Rheumatoid factor positive	−0.032	0.6
CDAI*	0.066	0.3
Deformed joints*	−0.076	0.3
Prednisone use	0.072	0.3
Erythrocyte sedimentation rate*	−0.066	0.4
C-reactive protein*	0.082	0.2
Interleukin-6*	0.067	0.3
Leukocytes*	0.001	1.0
Alanine aminotransferase*	0.043	0.5
Aspartate aminotransferase*	0.011	0.9
Glomerular filtration rate*	**−0.134**	**0.05**

Data were analyzed in mixed regression models in which sex and race, age at study time and race, age at study time and sex, and all 3 demographic characteristics were adjusted for in the 1^st^ and 2^nd^, 3^rd^, 4^th^ and the remaining models, respectively. Significant associations are shown in bold type. RA  =  rheumatoid arthritis, CDAI  =  Clinical Disease Activity Score.

*Logarithmically transformed variables.

### Independent associations of RBP4 concentrations with metabolic risk

Table 3 shows that RBP4 concentrations were independently and inversely associated with systolic and mean blood pressure, and glucose concentrations. RBP4 concentrations were not related to lipid values, levels of other adipokines and the leptin-adiponectin ratio.

**Table 3:** Independent relationships of retinol binding protein 4 concentrations with metabolic risk factors.

Characteristics	Partial R	p
Conventional metabolic risk factors		
Blood pressure value		
Systolic blood pressure	**−0.207**	**0.003**
Diastolic blood pressure	−0.102	0.2
Mean blood pressure	−**0.195**	**0.006**
Lipid values		
Total cholesterol	0.090	0.2
HDL cholesterol*	−0.002	1.0
Total cholesterol-HDL cholesterol ratio	0.060	0.2
LDL cholesterol	0.089	0.2
Non HDL cholesterol	0.099	0.2
Triacylglycerols*	−0.031	0.7
Triacylglycerols-HDL cholesterol ratio*	−0.022	0.8
Glucose*	−**0.155**	**0.03**
**Adipokines**		
Leptin*	0.052	0.5
Adiponectin*	−0.044	0.6
Leptin-adiponectin ratio*	0.043	0.6
Resistin	0.024	0.7

Data were analyzed in mixed regression models in which demographic characteristics, alcohol use, the waist-hip ratio, the glomerular filtration rate and cardiovascular drugs were adjusted for. Significant associations are shown in bold type. HDL  =  high-density lipoprotein, LDL  =  low-density lipoprotein, VCAM  =  vascular adhesion molecule, ICAM  =  intercellular adhesion molecule, MCP  =  monocyte chemoattractant protein. *Logarithmically transformed variables.

Table 4 gives findings in sensitivity analysis. RBP4 concentrations were associated with systolic and mean blood pressure in hypertensive but not in normotensive patients with RA. In patients with raised fasting glucose [31], the RBP4-glucose relation was numerically stronger than in the whole group (partial R = −0.184 versus −0.155 (see Table 3), but this was not nevertheless significant, possibly because of the small number of patients in this group (n = 34). RBP4 concentrations were not associated with those of glucose in patients with normal fasting glucose levels.

**Table 4:** Independent relationships of retinol binding protein 4 concentrations with systolic and mean blood pressure, and glucose levels amongst subgroups.

Relationship	Subgroups	Partial R	p
Retinol binding protein 4 versus			
Systolic blood pressure			
	Hypertensives (n = 135)	−**0.213**	**0.02**
	Normotensives (n = 82)	−0.028	0.8
Mean blood pressure			
	Hypertensives (n = 135)	−**0.209**	**0.02**
	Normotensives (n = 82)	0.011	0.9
Glucose*			
	Glucose ≥5.6 mmol/l (n = 31)	−0.184	0.4
	Glucose <5.6 mmol/l (n = 181)	−0.026	0.8
	Missing data (n = 5)		

Data were analyzed in adjusted mixed regression models as in Table 3. Significant differences amongst relations are shown in bold type. *Logarithmically transformed variable.

### Independent relationships of RBP4 concentrations with endothelial activation and carotid atherosclerosis

As shown in Table 5, RBP4 concentrations were independently and inversely related to those of E-selectin; the positive relation between RBP4 and MCP-1 did not reach significance (p = 0.06). RBP4 concentrations were not associated with cIMT and plaque.

**Table 5:** Independent relationships of retinol binding protein 4 concentrations with endothelial activation and atherosclerosis.

Characteristics	Partial R	p
Endothelial activation molecules		
E-selectin	−**0.191**	**0.007**
VCAM-1*	0.012	0.9
ICAM-1*	−0.039	0.6
MCP-1*	0.135	0.06
Carotid atherosclerosis		
Intima-media thickness	−0.000	1.0
	**OR (95% CI)**	**p**
Plaque	1.18 (0.86–1.61)	0.3

Retinol binding protein 4-endothelial activation and retinol binding protein 4-atherosclerosis relations were assessed in mixed regression models with adjustment for the log Framingham score (calculated from age, sex and major conventional risk factors), race, alcohol use, waist-hip ratio, glomerular filtration rate, and systemic inflammation (C-reactive protein and interleukin-6 concentrations). Significant association is shown in bold type.

VCAM  =  vascular cell adhesion molecule, ICAM  =  intercellular adhesion molecule, OR  =  odds ratio, CI  =  confidence interval.

*Characteristics that were non-normally distributed and were logarithmically transformed prior to entering them in linear regression models.

Table 6 gives the results obtained in sensitivity analysis. The inverse RBP4 - E-selectin relation was reproduced in patients aged ≤55 but not > 55 years, and in those with but not without ≥2 major traditional cardiovascular risk factors, a Framingham score of ≥2, overall and abdominal obesity, an RA duration of >10 years, absent or mild disease activity, an erythrocyte sedimentation rate of >12 mm/hr and black ethnicity; RBP4 concentrations were not significantly associated with those of E-selectin in patients with and without carotid artery plaque. The extent of effect of RBP4 concentrations on those of E-selectin was larger in those with compared to those without ≥2 major cardiovascular risk factors (standardized β (SE)  =  −0.534 (0.174) versus −0.151 (0.081), p = 0.04).

**Table 6:** Independent relationships of retinol binding protein 4 concentrations with those of E-selectin and atherosclerosis amongst subgroups.

	E-selectin		cIMT		Plaque	
Subgroups	Partial R	p	Partial R	p	OR (95% CI)	p
Age >55						
Yes (n = 127)	−0.140	0.1	−0.067	0.5	0.89 (0.61–1.29)	0.5
No (n = 90)	**−0.246**	**0.03**	**−**0.016	0.9	0.89 (0.45–1.75)	0.7
≥2 major traditional CV risk factor						
Yes (n = 58)	**−0.440**	**0.004**	0.125	0.4	1.55 (0.66–3.65)	0.3
No (n = 151)	**−**0.154	0.07	**−**0.030	0.7	1.09 (0.75–1.57)	0.7
Missing data (n = 8)						
Framingham score **>**2						
Yes (n = 121)	**−0.258**	**0.006**	**−**0.036	0.7	1.07 (0.71–1.60)	0.7
No (n = 88)	**−**0.115	0.3	**−**0.154	0.2	0.89 (0.49–1.59)	0.7
Missing data (n = 8)						
Carotid artery plaque						
Yes (n = 88)	**−**0.178	0.1	0.108	0.4	….	….
No (n = 129)	**−**0.181	0.054	**−**0.060	0.5	….	….
Obesity						
Yes (n = 107)	**−0.282**	**0.04**	**0.297**	**0.03**	**2.95 (1.31–6.68)**	**0.008**
No (n = 104)	**−**0.108	0.2	**−**0.113	0.2	0.71 (0.48–1.05)	0.08
Missing (n = 6)						
MetS waist						
Present (n = 100)	**−0.231**	**0.03**	0.016	0.9	**1.95 (1.12–3.42)**	**0.01**
Absent (n = 114)	**−**0.181	0.07	**−**0.048	0.6	0.65 (0.42–1.01)	0.06
Missing data (n = 3)						
RA duration >10 years						
Yes (n = 116)	**−0.236**	**0.01**	**−**0.015	0.9	1.09 (0.72–1.66)	0.7
No (n = 100)	**−**0.133	0.3	0.063	0.6	1.04 (0.60–1.83)	0.9
Missing data (n = 1)						
CDAI >10						
Yes (n = 92)	**−0.247**	**0.02**	0.093	0.4	1.01 (0.64–1.62)	0.9
No (n = 125)	**−**0.158	0.1	0.008	0.9	1.05 (0.69–1.60)	0.8
Erythrocyte sedimentation rate >12 mm/hr						
Yes (n = 112)	**−0.220**	**0.03**	**−**0.045	0.7	1.32 (0.86–2.03)	0.2
No (n = 99)	**−**0.170	0.1	0.002	1.0	0.78 (0.44–1.41)	0.4
Missing data (n = 6)						
Population						
Black (n = 112)	**−0.228**	**0.02**	0.149	0.1	**1.74 (1.06–2.86)**	**0.02**
White (n = 105)	**−**0.170	0.09	**−**0.145	0.2	0.85 (0.52–1.40)	0.5

Data were analyzed in adjusted mixed regression models as in Table 5. Significant association is shown in bold type. cIMT  =  carotid intima-media thickness, OR  =  odds ratio, CI  =  confidence intervals, CV  =  cardiovascular, MetS  =  metabolic syndrome, RA  =  rheumatoid arthritis, CDAI  =  Clinical Disease Activity Score.

As also shown in Table 6, RBP4 concentrations were associated with cIMT and plaque in patients with but not without overall obesity, and with plaque in those with but not without abdominal obesity. Further, RBP4 concentrations were independently related to plaque in black but not white patients with RA.

When RA characteristics were additionally adjusted for in the models in Tables 5 and 6, the results were materially unaltered (data not shown).

The mean (SD) RBP4 concentrations were numerically but not significantly higher in those with and without established cardiovascular disease (2.39 (1.28) versus 1.77 (0.94) uM/l, p = 0.1). In view of the small number of patients with the respective outcome characteristic, multivariable analysis was not performed.

## Discussion

In this RA study, adiposity was not associated with RBP4 concentrations. However, obesity adversely influenced the impact of this adipokine on atherosclerosis. Indeed, RBP4 concentrations were independently related to increased carotid atherosclerosis only in patients with overall and abdominal obesity. Further, although RBP4 concentrations were lower in black compared to white patients, they were associated with atherosclerosis in the former but not the latter. This was possibly due to the far larger prevalence of excess adiposity in black compared to white patients. Consideration of RBP4 concentrations may improve cardiovascular risk stratification in RA [18].

Paradoxical adipokine - CVD risk associations as we found between RBP4 concentrations and metabolic risk and endothelial activation, were previously reported [25], [43]. Such relations are thought to represent a compensatory change in adipokine production in the presence of chronic vascular disease and aimed at reducing metabolic risk [43]. However, we found that the presence of plaque did not influence the impact of RBP4 concentrations on cardiovascular risk. By contrast, the inverse RBP4 - CVD risk associations were mostly reproduced only in patients with adverse traditional or nontraditional cardiovascular risk profiles. Our cross-sectional study design precludes drawing inferences on the direction of causality. Additionally, our findings on the associations of RBP4 concentrations with metabolic risk factors and endothelial activation differ and in fact contrast to those previously reported in non-RA subjects [5], [9]. Therefore, these relations need further study.

RBP4 induces insulin resistance [44]. We did not directly assess this metabolic risk factor. RBP4 concentrations were nonetheless not associated with the triacylglycerols-HDL cholesterol and leptin-adiponectin ratios, which are surrogate markers of insulin resistance [45], [46]. Future studies on the potential impact of RBP4 on metabolic risk in RA should include markers of insulin resistance such as the homeostasis model assessment of insulin resistance.

Our finding of lower RBP4 concentrations in black compared to white patients with RA is congruent with an earlier investigation that showed disparities in RBP4 gene polymorphisms amongst black and white Americans [6]. Although increased liver fat content [8] is associated with increased RBP4 concentrations, we are unaware of a reported relation of alcohol consumption with levels of this adipokine, as shown by us in RA. Importantly also in this context, none of the reported independent associations between RBP4 concentrations and other recorded characteristics in the present study were altered upon further adjustment for alanine and aspartate aminotransferase concentrations (data not shown). Aminotransferase levels were previously shown to independently relate to metabolic risk and atherosclerosis in RA [47].

We assessed the production of 4 endothelial activation molecules that mediate the initial stages of atherosclerosis. Endothelial activation is strongly up-regulated and associated with prevalent and incident atherosclerosis in RA [48], [49]. We comprehensively adjusted for a wide range of confounders including the anthropometric measure of waist-hip circumference that is associated with RBP4 concentrations and cardiovascular events in non-RA subjects. Anthropometric measures are also related to atherosclerosis in RA [35]. This study has however additional limitations. Retinol and iron status are further potential confounders in the present context and were not evaluated [5]. Micronutrient deficiencies exist in urbanized South Africans from which our participants originated [50]. In a recent national survey on 3229 adult South Africans (79% black), retinol intake was documented to be adequate and iron intake inadequate [50]. Also, the effects of RBP4 on endothelial activation occur independently of retinol [9]. As was done in the original study by Graham and colleagues [3] as well as in many other reported investigations that followed, RBP4 concentrations were measured by ELISA, which can undervalue RBP4 concentrations in insulin resistant persons [5], [51]. Quantitative western blotting standardized to full-length RBP4 protein is suggested to be the most reliable method to measure RBP4 levels [5], [51]. Nevertheless, in an investigation by von Eynatten and colleagues [52], RBP4 concentrations quantified by ELISA were strongly associated with those measured by western blotting (R = 0.70 to 0.92). Finally, circulating RBP4 concentrations do not necessarily represent its tissue concentrations [5].

## Conclusion

The present study reveals that RBP4 concentrations are inversely related to metabolic risk and endothelial activation in RA. These findings require further elucidation. RBP4 concentrations were independently associated with enhanced atherosclerosis in RA patients with generalized or/and abdominal obesity.
